# II–VI Semiconductor-Based Conductometric Gas Sensors: Is There a Future for These Sensors?

**DOI:** 10.3390/s24123861

**Published:** 2024-06-14

**Authors:** Ghenadii Korotcenkov

**Affiliations:** Department of Physics and Engineering, Moldova State University, Str. Mateevici 60, MD-2009 Chisinau, Moldova; ghkoro@yahoo.com; Tel.: +373-60-642-109

**Keywords:** ZnS, CdS, ZnSe, CdSe, properties, advantages, performances, optimization, surface, stability, oxidation, limitations

## Abstract

A review of the state of research in the development of conductometric gas sensors based on II–VI semiconductors is given. It was shown that II–VI compounds indeed have properties that are necessary for the development of highly efficient gas sensors. In this case, to achieve the required parameters, all approaches developed for metal oxides can be used. At the same time, during a detailed review, it was concluded that sensors based on II–VI compounds have no prospects for appearing on the gas sensor market. The main obstacle is the instability of the surface state, which leads to poor reproducibility of parameters and drift of sensor characteristics during operation.

## 1. II–VI Semiconductor Compounds and Their Prospects for Gas Sensor Applications

II–VI semiconductor compounds, such as ZnS, ZnSe, CdS, CdSe, etc., are stable materials with high photosensitivity and good luminescent characteristics. In addition, for synthesis and deposition of these semiconductors, all dry and wet chemical methods developed for other semiconductor materials, including metal oxides, can be used. In particular powders, nanocrystals and quantum dots (QDs) of II–VI compounds can be synthesized using sol–gel technology; hydrothermal, solvothermal and microwave-assistant methods; precipitation; spray pyrolysis; aerosol flow synthesis; etc. Thin single and polycrystalline films can be formed using methods of both thin-film and thick-film technologies, including dip coating, screen printing, spin coating, spray coating, inkjet printing, vacuum thermal evaporation, magnetron sputtering, spray pyrolysis, pulse laser deposition, epitaxial growth, chemical vapor deposition (CVD), deposition in a chemical bath, electrochemical deposition, and some other methods that have been actively developed in recent years. These methods, as applied to II–VI compounds, are described in sufficient detail in [1]. Therefore, these materials are very useful in the development of thin-film solar cells, radiation detectors, photocatalysts, various optoelectronic devices, photoresistors, LEDs, and phosphors [2,3,4,5,6,7]. However, it turned out that II–VI semiconductor compounds, in addition to having excellent luminescent and photoelectric properties, also have interesting surface properties important for gas sensor applications [8,9]. Some parameters of compounds II–VI, important for applications, are given in Table 1. Other parameters of II–VI compounds can be found in [10,11,12,13,14]. In [1,2,3,4,5,6,7], you can also find a description of the approaches used in the manufacturing of II–VI semiconductor-based devices, including gas sensors.

**Figure 1:**
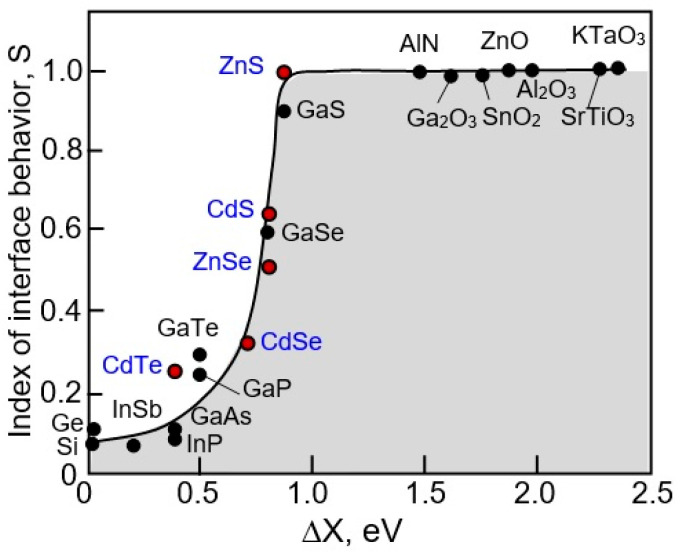
Influence of electronegativity on the value of *S* in φ_Bn_ = *S*(Δ*X*) dependence, where φ_Bn_ (eV) is the height of the Schottky barrier, and Δ*X* (eV) is the difference in electronegativity (*X*) of the metal and semiconductor. Adapted with permission from [23]. Copyright 1969 American Physical Society.

It is generally accepted that semiconductors can be divided into two broad groups based on the properties of the semiconductor–metal interface. “Ionic” materials are characterized by the absence of Fermi level pinning at the metal–semiconductor interface, while “covalent” materials demonstrate almost complete Fermi level pinning. Figure 1 illustrates this statement. In particular, the slope S of the dependence φ_Bn_ = S(ΔX), which inversely correlates with the degree of the Fermi level pinning, turned out to be equal to 1.0 (no Fermi-level pinning) for “ionic” materials and 0.1 (almost complete Fermi level pinning) for “covalent” materials. Metal oxides are representatives of “ionic” materials for which S = 1.0. The absence of the Fermi level pinning is explained by the low density of surface states (<10^11^ cm^−2^) in such materials. That is why the main materials for the development of conductometric gas sensors are conducting metal oxides, such as SnO_2_, In_2_O_3_, TiO_2_, ZnO, WO_3_, and Ga_2_O_3_, which, in addition to the absence of Fermi level pinning, are also stable in an oxygen atmosphere [15]. The good stability of metal oxides allows them to work in aggressive environments, and the absence of pinning of the surface Fermi level provides the maximum range of changes in the surface potential when interacting with a gas environment, necessary to achieve maximum sensory response. This is why covalent semiconductors such as GaAs, InP, GaP, Si, and Ge, for which S is in the order of 0.1–0.2, i.e., the surface Fermi level is firmly pinned within their band gap due to the high density of surface states, are practically not used in conductometric gas sensors. A more detailed description of the mechanisms of interaction of gases with the surface of gas-sensitive materials, explaining the appearance of the conductometric response, can be found in numerous reviews and books [16,17,18,19,20,21,22].

As can be seen in Figure 1, for II–VI compounds, the slope *S* varies from 0.3 for CdTe and CdSe to 0.6 for CdS and 1.0 for ZnS. This means that the position of the surface Fermi level, i.e., the band bending in these semiconductors, can also vary over a wide range, contributing to achieving a high sensor response when interacting with a gas environment. At the same time, wide-gap semiconductors ZnS and CdS have a maximum *S* value, which makes them most attractive for the development of solid-state conductometric gas sensors. It is important that the sensitivity of conductometric type gas sensors based on II–VI semiconductors for various compounds varies depending on the value of *S* from Figure 1. For example, in the CdS—CdSe pair, CdS-based sensors had much better sensitivity to water, ethanol, ammonia, acetone, and iodine vapors [24]. ZnS and CdS compounds, as well as metal oxides, are fairly stable materials in an oxygen atmosphere. It is important to note that what was said earlier is not unexpected, since metal oxides, such as ZnO and CdO, which are highly sensitive to gases [15], also belong to compounds of groups II-VI. It was also found that, like metal oxides, ZnS and CdS have non-stoichiometric surfaces [25,26].

**Figure 2:**
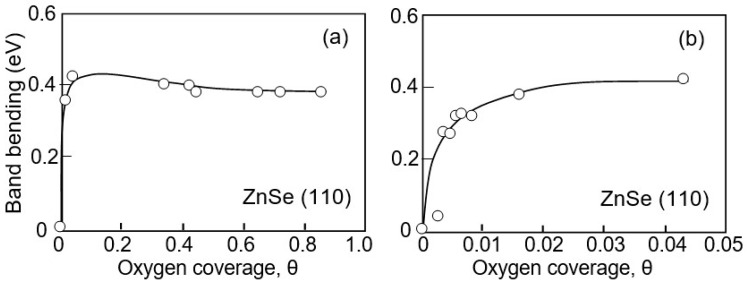
Changes in band bending ΔqV as a function of oxygen coverage for ZnSe(110); (**a**) in the region up to 0.83 monolayer oxygen and (**b**) in the region of small coverages where the changes are large. Data extracted from [25].

Many similarities in the behavior of metal oxides and II–VI compounds are also observed during their interaction with gas and water vapor [27,28,29]. It is shown that, as in the case of metal oxides, the interaction of II–VI semiconductors with molecules of toxic gases occurs through physisorption and chemisorption, which cause significant changes in the electronic properties of semiconductors, in particular the total and partial density of states and surface charge, which is a necessary condition to observe gas-sensitive effects in materials intended for conductometric gas sensors. In this case, different gases interact differently with the surface of II–VI semiconductors. For example, the interaction of CdS with SO_2_, H_2_S, and SO is stronger compared to CO and CO_2_. While CO_2_ and CO showed strong physical adsorption on the CdS layer, SO_2_, H_2_S, and SO molecules showed chemisorption with higher adsorption energies [27]. Just as for metal oxides, the surface Fermi level (*E*_F_) shifts towards the midgap with the adsorption of oxygen for II–VI semiconductors, which results in an upward band bend for n-type semiconductors (ZnS, CdS, and ZnSe) [25,30] and a downward band bend for p-type semiconductors (ZnTe) [25]. The influence of oxygen coverage on the band bending for n-ZnSe is shown in Figure 2. The appearance of a potential barrier on the surface is explained by the capture of electrons from the conduction band by chemisorbed oxygen. According to generally accepted concepts, it is the change in the surface charge captured by oxygen in the process of interaction with the detected gases that determines the sensor response of conductometric gas sensors [16,17,18,19,20,21,22].

**Figure 3:**
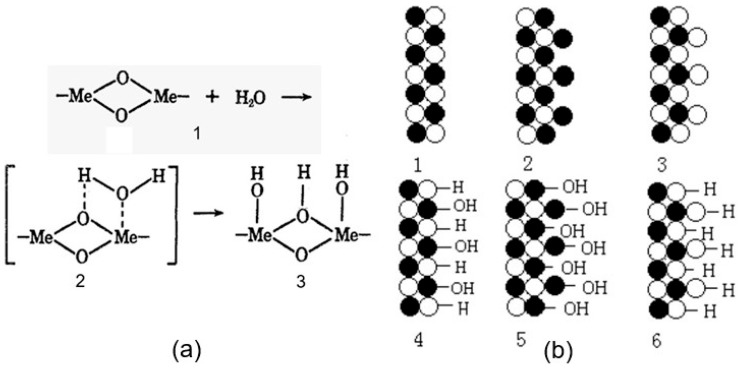
(**a**) Schematic diagram illustrating water adsorption on the surface of metal oxides: At the first stage of adsorption, a water molecule will be physically adsorbed on an activated site (1) to form an adsorption complex (2), which will subsequently transfer to surface hydroxyl groups (3). Adapted with permission from [35]. Copyright 1969: ACS. (**b**) Schematic illustration of the surface stoichiometry and hydration of II–VI semiconductor: 1—Non-hydrated stoichiometric II–VI surface; 2—Non-hydrated zinc (cadmium)-rich II–VI surface; 3—Non-hydrated sulfur (selenium, tellurium)-rich II–VI surface; 4—Hydrated stoichiometric II–VI surface, 5—Hydrated zinc (Cd)-rich II–VI surface; 6—Hydrated sulfur (Se, Te)-rich II–VI surface. Reprinted from [34]. Published 2011 by BMC as open access.

Just as in the case of metal oxides, the state of the surface of II–VI semiconductors determines the characteristics of adsorption–catalytic processes. According to this model, the active centers involved in these processes are predominantly coordinately unsaturated surface atoms and structural vacancy defects in II–VI compounds [31]. As shown by mass spectrometric studies, the main components of the desorbed phase from the surface of ZnSe, ZnTe, CdSe, and CdTe samples after interaction with the surrounding atmosphere are H_2_O, CO_2_, CO, and O_2_ [32]. In addition, hydrogen and reaction products are also found in small amounts. As the time of exposure to air increases, water, carbon dioxide, and oxygen accumulate predominantly on the surface of II–VI compounds. At the same time, at the early stages of adsorption, water plays the greatest part in the formation of surface properties. It also plays a decisive role in surface charging. Chemisorption of a water molecule on the surface of CdS was established by Gupta and van Huis [33]. Figure 3 shows how the surfaces of II–VI semiconductors behave when interacting with water. As can be seen from Figure 3b, water dissociates to form a proton and hydroxide, the concentration of which depends on the stoichiometry of the surface; the proton and hydroxide are balanced or one of them dominates at the surface [34]. It is precisely this mechanism that is realized on the surface of metal oxides (Figure 3a). In this case, there are three possible ways of binding water molecules to the surfaces of II–VI semiconductors: (a) through the formation of Zn(Cd)-O bonds; (b) due to the formation of S(Se,Te)-H bonds; and (c) due to the formation of Zn(Cd)-O and S(Se,Te)-H bonds. This means that surface stoichiometry strongly influences the surface properties of II–VI semiconductors; either a Zn (Cd)-rich surface or a S (Se, Te)-rich surface will have different acid–base properties, that is, different catalytic and gas sensing properties.

**Figure 4:**
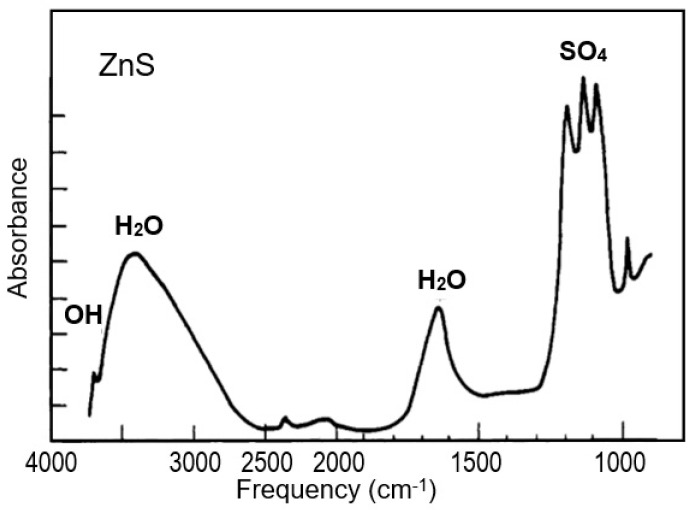
Fourier-transform infrared spectroscopy (FTIR) spectrum of ZnS powders. Adapted with permission from [31]. Copyright 1988: ACS.

The previously presented results of mass-spectrometric studies were confirmed and supplemented by the IR spectra of II–VI semiconductor films [31,36]. After exposure to air, they contain bands of stretching (3570–3450 cm^−1^) and bending (1650–1620 cm^−1^) vibrations of adsorbed water, a small number of absorption bands in the regions of 2960–2860 cm^−1^ and 1460–1380 cm^−1^, which correspond to vibrations of the C–H, C–O groups, as well as absorption bands around 1150 and 1205 cm^−1^, for which hydrated metal oxides may be responsible. According to IR spectra, thermal treatment of samples at T = 420–473 K in a helium flow leads to a decrease in the intensity of all absorption bands and predominantly of adsorbed water. At 250–400 °C, adsorbed impurities are almost completely removed. Figure 4 shows a typical spectrum of purified zinc sulfide. Bands of hydroxyl groups (3691 cm^−1^), water (3410, 1640 cm^−1^; some of which remain sorbed after evacuation at 400 °C), and sulfate groups (1086, 1132, and 1191 cm^−1^) are observed. ZnOH groups should not be expected to appear on a clean ZnS surface. This material is partially oxidized on the surface, as evidenced by the presence of sulfate groups.

An experiment with single crystals and polycrystalline materials showed that at low temperatures, the adsorption of gases is usually not accompanied by chemical reactions affecting the structure of the semiconductor. In particular, Campbell and Farnsworth [30] found that the adsorption of CO_2_, H_2_O, H_2_, and CH_4_ at room temperatures was not accompanied by a change in the diffraction patterns of a clean CdS surface. Although at the nanoscale, as in the case of metal oxides, the influence of water increases. Thus, Goodell et al. [37] observed a structural transformation of zinc sulfide nanoparticles with a diameter of 3 nm caused by the adsorption of water. Zhang et al. [38] also found that decreasing particle size affected water adsorption. They found that, firstly, ZnS nanoparticles (3 nm) can adsorb more water molecules per unit surface area, and secondly, the binding energy of water in nanoparticles is higher than in bulk crystals. Zhang et al. [38] believe that this finding can be extended to other nanoparticles that interact with various molecules present in the environment.

Just as in the case of metal oxides, the concentration of adsorbed molecules decreases with increasing temperature. With increasing temperature, a change in desorption activity is observed with maxima at 100–200 °C (for weakly bound oxygen and molecular water) and 300 °C (for chemisorbed water). Bootsma [32] studied the adsorption of oxygen and H_X_A gases (H_2_S, HBr, NH_3_, and PH_3_) on the surface of CdS and found that the amounts adsorbed at a given pressure for all gases are negligible at 400 °C. It follows that the removal of these gas impurities during thermal vacuum treatment must include at least two stages: 1—heating the samples to 180–200 °C and maintaining them at this temperature for several hours to more completely remove oxygen and molecular water, weakly attached to the surface; 2—further heating to remove chemisorbed water. As a result of this treatment, a certain amount of chemisorbed oxygen remains on the surface. According to Campbell and Farnsworth [30], chemisorbed oxygen cannot be removed at room temperature. It has been established that the rate of oxygen desorption from the surface of CdS in a vacuum depends exponentially on the reciprocal of the absolute temperature, so that to remove a monolayer of oxygen it takes 4 h at 350 °C or 5 min at 550 °C. In an argon atmosphere, chemisorbed oxygen was almost completely removed as a result of the heat treatment of single-crystal samples at T ~700 °C. As we can see, the desorption of gas impurities adsorbed on metal oxides and the surfaces of II–VI semiconductors occurs in almost the same temperature ranges.

All of the above indicates that II–VI semiconductors, in their properties, in many cases, are analogues of metal oxides. This similarity explains why many attempts have been made in recent years to develop conductometric gas sensors based on them [39,40,41,42,43,44]. In addition, it has been established that II–VI semiconductor compounds have other properties that are important for gas-sensitive materials intended for use in conductometric gas sensors [45]: (i) semiconductors have acceptable conductivity; (ii) the band gap is sufficient to operate at elevated temperatures; and (iii) the surface has the properties necessary for effective interaction with the target gas. Other advantages of II–VI compounds also include their fairly good processability, which allows for (1) the formation of structures with high gas permeability to achieve high sensitivity and fast response, (2) the synthesis of single crystals and powders, and (3) the formation of epitaxial and polycrystalline films with specified parameters using different precipitation and deposition methods [43]. Moreover, like metal oxides, II–VI semiconductors can be synthesized as 1D, 2D, and 3D nanostructures [41]. The synthesis of quantum dots (QDs) based on II–VI semiconductors is also possible [46,47]. All of the above technological capabilities make it possible to develop conductometric gas sensors based on II–VI semiconductors using various sensor platforms [47,48].

## 2. Current Status

**Figure 5:**
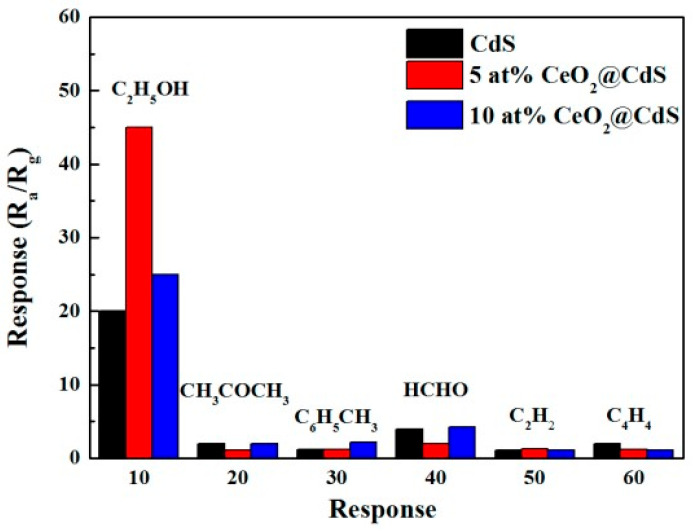
Selectivity of the sensors to 100 ppm ethanol (C_2_H_5_OH), acetone (CH_3_COCH_3_), toluene (C_6_H_5_CH_3_), formaldehyde (HCHO), ethyne (C_2_H_2_), and tetrahedrane (C_4_H_4_). Reprinted from [50]. Published 2017 by MDPI as open access.

**Figure 6:**
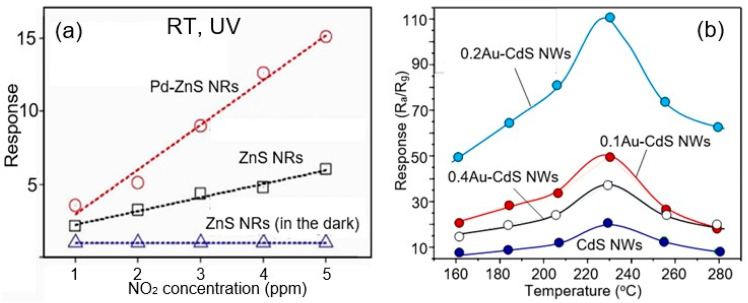
(**a**) Responses at RT of the pristine ZnS nanorod gas sensor in the dark and under UV illumination, and the Pd-functionalized ZnS nanorod gas sensor under UV illumination as a function of the NO_2_ gas concentration. ZnS nanorods were synthesized by the thermal evaporation of ZnS powders. A thin Pd film was deposited onto the surfaces of the ZnS nanorod samples using a wet method. Sensors were fabricated using drop coating of ZnS NRs. Adapted with permission from [74]. Copyright 2014: Elsevier. (**b**) Responses of the CdS sensors to 100 ppm ethanol at different operating temperatures. CdS NWs were synthesized by a typical one-step solvothermal method. The different dosage of HAuCl_4_ (0.1 mL, 0.2 mL, and 0.4 mL) was added in the procedure of Au-nanoparticle decoration and the corresponding Au nanoparticle-decorated CdS NWs were denoted as 0.1Au–CdS NWs, 0.2Au–CdS NWs, and 0.4Au–CdS NWs, respectively. The sensor response was calculated as R_a_/R_g_, where R_a_ is the sensor resistance measured in the atmosphere and R_g_ is the sensor resistance measured in the test gas atmosphere. Adapted with permission from [75]. Copyright 2016: RSC.

**Figure 7:**
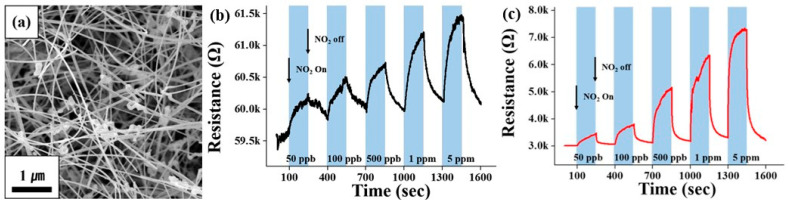
(**a**) SEM image of ZnSe nanowires. The 1D nanostructures exhibited a wire- or fiber-like morphology with widths ranging from 30 to 100 nm and lengths ranging up to ≈300 μm. (**b**,**c**) Conductometric responses of the gas sensors fabricated from ZnSe nanowires to 50 ppb, 100 ppb, 500 ppb, 1 ppm, and 5 ppm NO_2_ gas at room temperature (**a**) in the dark and (**b**) under UV (365 nm) illumination at 1.2 mW/cm^2^. Reprinted from [49]. Published 2014 by Beilstein-Institut as open access.

Experimental studies confirmed the conclusions made earlier. Conductometric gas sensors based on ZnS, CdS, ZnSe, and CdSe, with high sensitivity to various gases can indeed be created. Currently, a fairly large number of articles and reviews have been published with research results in this area [29,39,40,41,42,43,44,49]. The main results of these studies can be summarized as follows:

- First, gas sensors based on II–VI semiconductors are sensitive to toxic and flammable gases and vapors. There are currently reports of the development of II–VI compound-based gas sensors capable of detecting O_2_, O_3_, SO_2_, H_2_, H_2_S, NH_3_, NO_2_, Cl_2_, CCl_4_, CO_2_, ethanol, methanol, acetone, benzene, formaldehyde, isopropanol, gasoline, toluene, and liquefied petroleum gas (LPG) [37,41,50]. Moreover, these sensors demonstrated sensitivity to these gases, acceptable for real use. As a rule, among the most dangerous gases, in most cases, the sensors had a maximum response to H_2_S [51,52] or NO_2_ [53,54], and among organic solvents, sensitivity decreased when moving from ethanol to methanol and acetone (formaldehyde, benzene, toluene, and acetylene) [39,50,55]. One example illustrating this behavior of sensors is shown in Figure 5. Of course, sensors developed in different laboratories could demonstrate completely different characteristics. For example, ZnS sensors developed by Shinde et al. [56], among toxic gases, had the maximum sensitivity to Cl_2_, and in the CdS sensors developed by Fu et al. [57] and Liu et al. [58] among organic solvents, isopropanol had the highest sensitivity.
- Secondly, it was found that the gas-sensitive mechanism [16,17,19] and all the regularities established for metal oxides [59] also work in gas sensors based on II–VI compounds [39,43,60]. This means that (i) for better sensitivity, films based on II–VI semiconductors should be porous with small grain size and a high specific surface area [61], (ii) reducing the film thickness and increasing the pore size helps reduce response and recovery time [62,63], and (iii) increasing operating temperature reduces response and recovery time [62]. As for the effect of film thickness on the sensory response, it depends on the type of semiconductor, film structure, and the gas being detected. For example, studies of CdSe and CdS films have demonstrated an increase in sensitivity to oxygen and carbon monoxide as the film thickness of these materials increases [62,63,64]. At the same time, Dzhurkov et al. [65] reported that their thermal sputtered ZnSe sensors showed a decrease in sensitivity to ethanol as the thickness increased from 50 to 160 nm. It was also found that the gas sensitivity of II–VI semiconductors showed a dependence of the gas sensor response on the crystal shape [58], as was previously found for metal oxides [66].

- Third, bulk doping and surface modification approaches developed for metal oxide gas sensors [67,68,69] can also be used to improve the selectivity and sensitivity of II–VI semiconductor-based sensors [43]. For example, doping CdS with zinc has been shown to increase the sensitivity of sensors to reducing gases such as methanol, ethanol, methylbenzene, formaldehyde, and acetone [70]. Something similar occurs when doping CdS with Ni^2+^ ions, which has been shown to improve the sensitivity of CdS to LPG [71]. Doping ZnS with Mn^2+^ ions also resulted in improved oxygen sensitivity [72]. Yue et al. [69] have shown that CdS doping with 1 mol%SnO_2_ strongly increases the sensitivity to ethanol. Decorating ZnS with Au nanoparticles enhanced the response to various gases (including nitrogen dioxide, ammonia, ethanol, carbon monoxide, and hydrogen sulfide) at room temperature compared to the equivalent system without nanoparticle decoration [73]. Decoration of CdS with Au nanoparticles increased the sensitivity to ethanol (Figure 6b). A similar study on palladium-decorated ZnS nanoparticles also showed a significant improvement in sensitivity to nitrogen dioxide compared to undecorated ZnS under UV illumination [74]. The results of this study are shown in Figure 6a.

- Fourth, an approach based on the use of nanostructures such as 0D (quantum dots), 1D (nanowires, nanotubes, and nanofibers), 2D (nanosheets and nanobelts) and 3D (hierarchical structures such as nanoflowers and hollow nanospheres) for the optimization of sensor parameters is also suitable for the development of sensors based on II–VI semiconductors [41,49]. For example, CdS QDs with their large specific surface area and good charge transfer properties led to the sensitization of sensors based on Co_3_O_4_ microspheres to H_2_S [76]. The CdS QDs/Co_3_O_4_ composites also showed very short response and recovery times at room temperature. The CdSe QD/In_2_O_3_ structure showed a better sensor response to NO_2_ compared to other composites [77]. ZnSe nanowire-based sensors developed by Park et al. [49] were also sensitive to NO_2_. Due to the specific properties of ZnSe nanowires (see Figure 7a), the sensors were able to detect NO_2_ at room temperature (Figure 7b). Of course, the sensitivity to NO_2_ at room temperature was low but reproducible. In this case, UV irradiation (365 nm) increased the response of ZnSe nanowires to a NO_2_ concentration of 50 ppb–5 ppm by 1.1–2.3 times (Figure 7c). It is known that light at room temperature can activate chemoresistance in II–VI semiconductors. It is this effect that in many cases allows gas sensors based on metal oxides and II–VI semiconductors to operate effectively at room temperature. However, the influence of environmental conditions on the parameters of semiconductor QDs and II–VI nanowires, especially their stability, limits the use of semiconductor QDs and II–VI nanowires in gas sensors operating at elevated temperatures [49]. For example, as will be shown later, CdS QDs can easily oxidize and transform into CdO at temperatures above 25 °C [76]. The same effect is typical for nanowires [49].

Research has shown that gas sensors designed using one-dimensional (1D) II–VI semiconductor nanostructures are sensitive to the same gases as sensors based on polycrystalline materials. At the same time, no improvement in parameters was observed, since the array of 1D nanostructures is not fundamentally different from a porous polycrystalline film [78]. As with polycrystalline sensors, the maximum sensory response was observed at a temperature of 200–300 °C [73,79,80].

**Figure 8:**
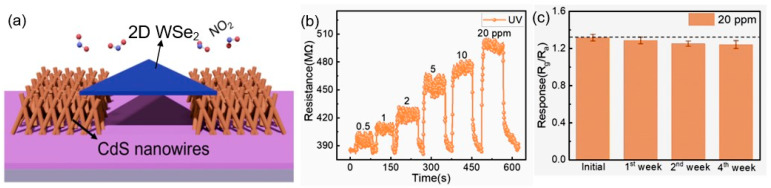
(**a**) Scheme of the CdS-WSe_2_ heterojunction-based sensor exposed to NO_2_. To fabricate the hybrid junction device, the WSe_2_ nanosheets were transferred to the patterned CdS nanowires arrays by the wet transfer method. (**b**) Dynamic sensing performance to various NO_2_ concentrations under UV illumination. (**c**) Long-term stability of the gas sensor to 20 ppm NO_2_. Dashed line corresponds to the initial value of the sensory response. Reprinted from [83]. Published 2024 in ChemRxiv as open access.

Two-dimensional nanostructures have been used less frequently in the development of sensors based on II–VI semiconductors [81,82]. Li et al. [82] presented a highly ordered CdS nanoflake array fabricated by CVD and grown directly on the gas sensor platform. The room temperature sensing performance of the fabricated sensors showed high sensitivity to 5 ppm NO_2_ under green LED illumination. In addition, sensors demonstrated good selectivity and small interference from humidity. Zheng et al. [83] showed that 2D p-type WSe_2_ nanosheets in combination with 3D n-type CdS nanowire arrays allow for the formation of a unique configuration (see Figure 8a) with a highly selective and reversible response to NO_2_ and NH_3_ with detection limits of 60 and 54 ppb, respectively, under UV illumination at room temperature (Figure 8b). The sensor exhibited an ultrafast response time of less than 1s to 1 ppm NO_2_ and NH_3_, which outperforms most previous reports on NO_2_ and NH_3_ detection at room temperature. Zheng et al. [83] believe that the outstanding sensing performance is attributed to the combination of tuning of the Schottky barrier at the n-CdS nanowire–p-WSe_2_ nanosheet heterojunction, the photoelectric effect, and mixed dimensionally contact mode. However, it should be recognized that to fabricate devices based on 2D nanomaterials, especially with the configuration shown in Figure 8a, significant technological difficulties associated with the separation and manipulation of individual nanosheets must be overcome. The parameters of sensors with such a configuration will strongly depend on both the parameters of nanowires (diameter) and nanosheets (thickness), as well as the area of the nanosheet and its position on the surface of the CdS nanowire arrays. It is almost impossible to achieve the required reproducibility of the parameters of such sensors. In addition, if 2D nanomaterials consist of large nanosheets, then when using thick-film technology for the deposition of a sensitive layer, the formation of highly agglomerated 2D nanosheets is possible [84].

**Figure 9:**
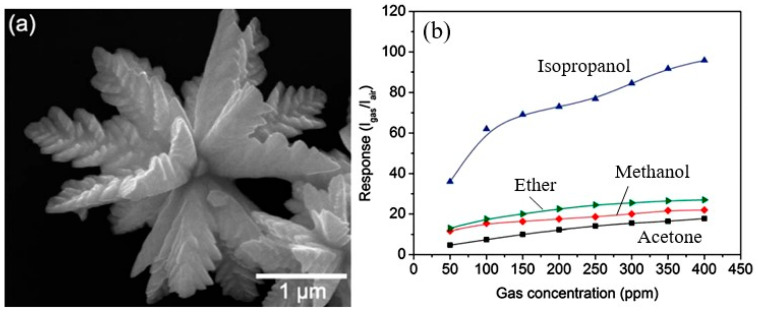
(**a**) Field emission SEM image of a leaf-like CdS micro-/nanostructure prepared by the hydrothermal method at 150 °C; and (**b**) calibration curves of CdS sensing responses vs. VOCs. The sensory response is calculated as I_gas_/I_air_, where I_air_ is the current through the sensor, measured in the atmosphere, and I_gas_ is the current through the sensor measured at the same voltage in the test gas atmosphere. Reproduced with permission from [57]. Copyright 2012: Royal Society of Chemistry.

Analysis of the results obtained from the study of gas sensors based on 3D nanostructures showed that the 3D nanostructure appears to be the most suitable configuration of a gas-sensitive material to achieve maximum sensitivity and fast response [41,84]. Such a structure can provide both a very high specific area of the active surface and high porosity and gas permeability with a minimum size of 0D, 1D, and 2D nanomaterials forming these 3D structures. Currently, based on II–VI compounds, a variety of 3D structures have been synthesized and used for the development of gas sensors. These 3D structures include hierarchical nanostructures [57,58,85,86], hollow spheres [87,88], and core/shell nanostructures [89,90,91]. These developments have shown that this direction is indeed very promising. For example, Fu et al. [57] synthesized a hierarchical CdS nanostructure (see Figure 9a) by the hydrothermal method and applied it to develop gas sensors. These sensors demonstrated outstanding detection performance, including high sensitivity, a low detection limit, and short response/recovery time for typical volatile organic compounds (VOCs), including ether, methanol, acetone, and isopropanol (Figure 9b). The results of their testing at an operating temperature of 210 °C showed their significant superiority in sensor performance compared to conventional CdS gas sensors.

**Figure 10:**
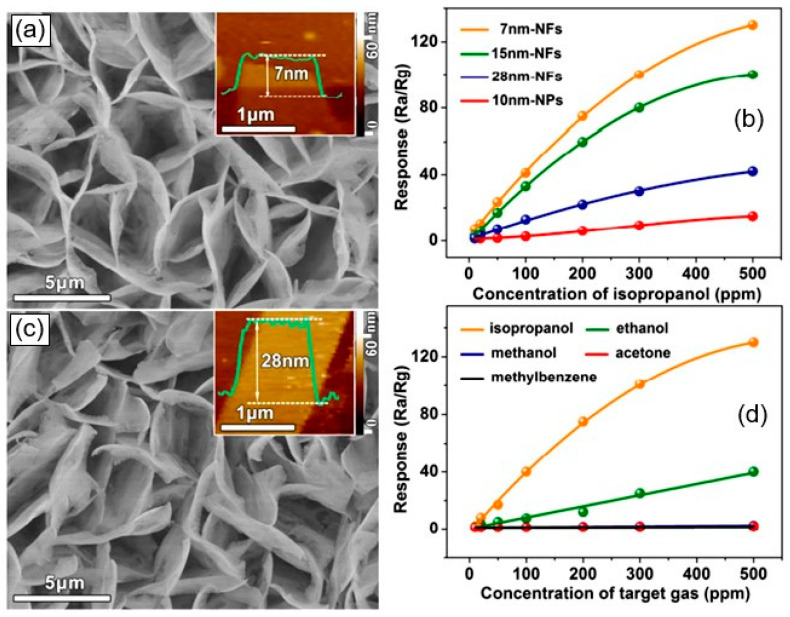
(**a**,**c**) SEM image of CdS nanoflakes (NFs) with different flake thickness: (**a**) 7 nm; (**c**) 28 nm (inset is an atomic force microscopy image of a nanoflake thickness). (**b**,**d**) Influence of the concentration of organic solvent vapors on the response of CdS NF-based sensors, fabricated on interdigitated electrode. Reproduced with permission from Liu et al. [58]. Copyright 2017: ACS Publications.

Liu et al. [58] proposed a different approach to the formation of 3D structures and the manufacture of gas sensors. It did not involve the use of thick-film technology methods. They synthesized a hierarchical CdS nanostructure, which is an ensemble of 2D nanostructures, directly on interdigitated electrodes using the CVD method (Figure 10a,c). The experiment showed that this structure of the gas-sensing layer provides many advantages in terms of gas detection, including the following: (i) it avoids the problem of stacking nanosheets on top of each other, and as a result, free access of the test gas to the entire surface is ensured and diffusion restriction in the sensory response is removed; (ii) it is possible to synthesize nanoflakes of ultra-thin thickness, which ensures the high sensitivity of the developed sensors. Indeed, the sensors developed in this way showed high selectivity towards isopropanol at 225 °C (Figure 10d), with better sensitivity performance compared to conventional CdS nanoparticle-based sensitive films.

**Figure 11:**
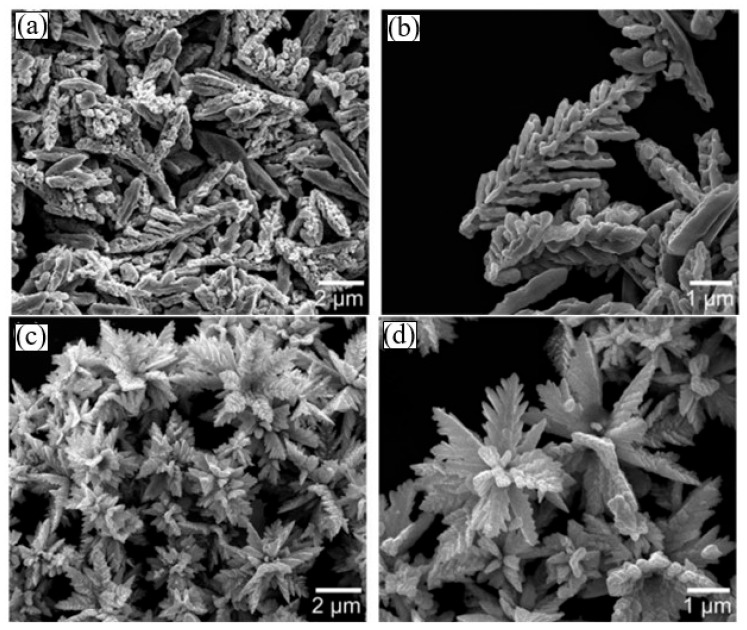
FESEM images of CdS samples prepared with different quantities of DMSO (C_2_H_6_SO) at 170 °C for 8 h: (**a**,**b**) 0 mL; (**c**,**d**) 0.2 mL. CdS nanostructures were prepared via a hydrothermal method. Reprinted with permission from [57]. Copyright 2012: Royal Society of Chemistry.

Regarding gas sensors developed based on hollow spheres and core/shell nanostructures, analysis of their test results showed that these sensors have high sensitivity and fast response [41,92,93,94]. For example, gas sensors with ZnSe/Cr-MoO_3_ nanospheres and ZnS@ZnO core/shell structures showed increased sensitivity to triethylamine (TEA) [92,95]. According to He et al. [93], sensors with SnO_2_@ZnS core/shell hollow spheres exhibited enhanced sensing properties to n-butanol at room temperature. They have shown that uniform core/shell hollow structures play critical roles in enhancing room-temperature sensing performances. However, it must be taken into account that when synthesizing 3D nanostructures with the required parameters, technological difficulties often arise [57], which reduce the reproducibility of the morphological parameters of the synthesized nanostructures, and hence the reproducibility of the parameters of sensors using these structures. For example, small changes in synthesis temperature, synthesis time, and solution composition used in the synthesis process result in the occurrence of significant morphological changes in three-dimensional structures (see Figure 10a,c and Figure 11). This means that the gas-sensitive characteristics of the sensors will inevitably also change (see Figure 10b).

- Fifth, just as for metal oxides, the use of heterostructures and nanocomposites under certain conditions can optimize sensor parameters. The experiment showed that, as in the case of metal oxides [96], the creation of inorganic nanocomposites based on II–VI semiconductors is one of the approaches that can significantly improve sensor performance (primarily sensitivity and selectivity, but in some cases also reduce power consumption) compared to sensors made on the basis of individual components forming a composite. For example, Table 2 presents the results of testing gas sensors based on the Zn_1−x_Cd_x_S nanocomposite. It can be seen that with the optimal composition (X = 0.4), there is an increase in the sensory response and a decrease in the response time and recovery compared to ZnS-based sensors. Zhu et al. [55] also showed that these sensors had less dependence on air humidity. Zhang et al. [97] synthesized CdS/WS_2_ composites by an environmentally friendly and ultra-low-cost hydrothermal method and found that, compared with pure WS_2_ and CdS, the CdS/WS_2–_40 wt% composite exhibits a superior sensor response of more than 4 orders of magnitude, very short recovery time (3 s), and ultra-high selectivity to NH_3_ at room temperature. Moreover, immunity tests showed that the CdS/WS_2_ sample remained stable under real-time NH_3_ monitoring at different ambient humidity levels.

**Figure 12:**
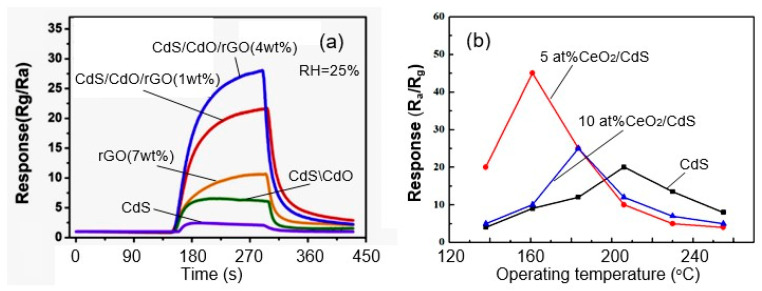
(**a**) The transient responses of CdS-based composites to 2 ppm NO_2_ at 125 °C. Reprinted with permission from [100]. Copyright 2019: Elsevier. (**b**) Response of CdS NWs and CeO_2_/CdS composites to 100 ppm ethanol vapor measured at different working temperatures from 138 °C to 255 °C. Reprinted from [50]. Published 2017 by MDPI as open access.

Currently, a large number of different nanocomposites have been synthesized and tested, including CdTe/ZnO, NiO/ZnSe, CdO/CdS, SnO_2_/ZnSe, ZnO/ZnSe, ZnS/TiO_2_, CuO/CdS, ZnS/In_2_O_3_, MoS_2_/n-CdS, SnS_2_/ZnS, ZnSe/Cr-MoO_3_, CdS/WS_2_, CeO_2_/CdS, CdS/TiO_2_, and others [77,92,94,97,98,99,100,101]. Features of the formation of nanocomposites and the performances of sensors based on them were considered by Vasiliev et al. [44]. It was found that, under optimal conditions, gas sensors based on nanocomposites with heterojunctions, including II–VI semiconductors, can provide (i) higher sensitivity due to control of the energy barrier at the interface of the contacting semiconductors, (ii) improved selectivity to certain gases, (iii) reduced sensitivity to interfering gases such as humidity, (iv) can shift the optical sensitivity of wide-band-gap metal oxide semiconductors to longer wavelengths to create sensors that operate at room temperature when activated by visible light, (v) improved performance due to improving film morphology, and (vi) reduced operating temperature. Examples of this behavior are shown in Figure 12.

The mechanisms of influence of the composition of nanocomposites on the parameters of sensors were considered in detail by Korotcenkov and Cho [96]. For example, according to Sun et al. [100], improvement in the parameters of sensors based on CdS/CdO/rGO nanocomposites is associated with the formation of heterojunctions between CdS and CdO, decorated with rGO, which acts as an effective electron mediator that increases gas adsorption and accelerates electron transfer in the composite. The same conclusion was reached by Tan et al. [92] when analyzing the characteristics of ZnSe/Cr-MoO_3_-based TEA sensors. The simulation analysis based on density functional theory (DFT) calculations indicated that the heterojunctions could effectively enhance the adsorption energy of TEA and there were more charges transferring from TEA to the Cr-MoO_3_ nanorods. Regarding CdS/CeO_2_ composites, Li et al. [50] believe that the reason for the improvement in sensitivity is that CeO_2_ acts as a catalyst for the oxidative dehydrogenation of alcohols. CeO_2_ nanoparticles on the CdS surface involve more ethanol molecules in the reaction by catalyzing them into acetaldehyde.

As shown by Kumar et al. [42], semiconductor–polymer nanocomposites can also be used to develop gas sensors based on II–VI compounds. Interest in such composites is due to the fact that by changing the amount and size of nanomaterials in a II–VI semiconductor–polymer composite, it is possible to achieve the increased sensitivity and selectivity of polymer gas sensors towards hazardous gases [102,103,104]. For example, based on this approach, a liquefied petroleum gas (LPG) sensor based on an n-CdSe/p-polyaniline (PANI) heterojunction [105], a gas sensor based on a CdSe/ZnS-polystyrene (PS) nanocomposite sensitive to chloroform, dimethylformamide (DMF), and tetrahydrofuran (THF) [106], and a sensor with excellent sensitivity to chloroform vapor at room temperature based on a CdSe/P3HT composite [107] were fabricated. It was also found that PEDOT:PSS/CdS nanocomposites exhibited enhanced sensitivity to LPG [108], the CdS-polypyrrole (PPY) nanocomposite demonstrated enhanced sensitivity to ammonia gas [109], CdS-polyvinyl alcohol (PVA) nanocomposites showed high sensitivity for NO_2_ gas at an operating temperature of 200 °C [110], and a nanocomposite-based resistive sensor PS/PMMA-CdSe/ZnS was characterized by an improved response to VOCs [111]. However, it should be noted that in these sensors, as a rule, II–VI semiconductors play only the role of a conductive additive that improves the electrical conductivity of nanocomposites and the porosity of the polymer matrix.

## 3. Limitations

**Figure 13:**
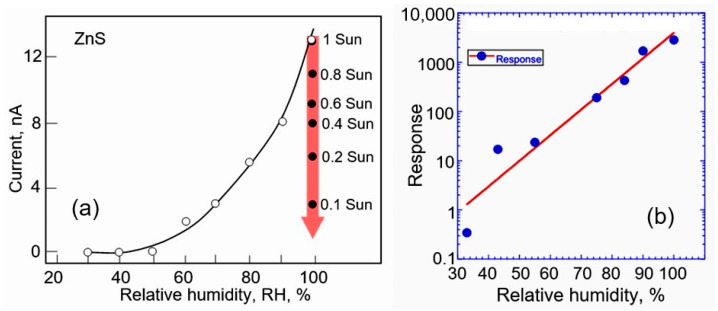
(**a**) Current measured in ZnS-based sensors in atmosphere with different RH under 1 sun illumination. In addition, this figure shows the influence of illumination on the current measured at RH 95%. Data extracted from [114]. (**b**) Variation in conductometric response of ZnS nanowires versus RH in the range of 22–97%. Reprinted with permission from [115]. Copyright 2012: Elsevier.

However, despite the previously presented results indicating the high gas sensitivity of II–VI compounds, it can be argued that gas sensors based on II–VI semiconductors have no prospects in the gas sensor market. That is, these sensors cannot replace metal oxide gas sensors that are available on the market and widely used [112]. There are several reasons for this:

✓ These compounds, unlike group III-V semiconductors, exhibit a greater tendency to deviate from stoichiometry, which allows them to be considered as phases of variable composition. Because of this, the properties of type II–VI semiconductor compounds remain difficult to control [12].
✓ The use of these materials does not provide improved performance compared to gas sensors based on metal oxides. Sensors based on II–VI semiconductors are sensitive to the same gases and vapors as metal oxide sensors. In addition, they, like metal oxide sensors, are strongly influenced by humidity, which leads to unreliable sensory responses, especially at high concentrations of water vapor [113]. How significant the effect of humidity is on resistive II–VI semiconductor-based sensors can be shown in Figure 11. It can be seen that in the region of high humidity levels, the resistance of ZnS nanowires can change hundreds of times (Figure 13b). Sensors based on II–VI compounds also have low selectivity [43]. For example, ZnS-based sensors preferentially respond to Cl_2_ [56]. However, they may experience high interference from CO_2_, H_2_S, and H_2_ and moderate interference from LPG, NH_3_, and ethanol (see Figure 3, Figure 9b and Figure 10d). This means that the application of II–VI semiconductor compounds cannot solve the problems of low selectivity of conductometric gas sensors based on metal oxides. Moreover, as a rule, the parameters of gas sensors based on II–VI semiconductors are significantly worse than the parameters of sensors based on metal oxides.

✓ The parameters of semiconductor compounds II–VI strongly depend on illumination (see, for example, Figure 13a) and temperature, which makes the choice of operating conditions for these sensors more stringent.
✓ The too high resistance of ZnS and CdS also creates difficulties in the design of gas sensors; this complicates the communication process with the measuring equipment [116]. For example, the sensors developed by Choudhari et al. [117] had a resistance greater than 10^9^ Ω.
✓ But the most important limitation is still the lack of temporal stability of these sensors, especially at elevated temperatures, when there is a drift in the baseline resistance and a decrease in the sensory response over time [9]. As shown in Figure 8c, a temporary change in sensory response is observed even when operating at room temperature.

It has been established that, despite high melting temperatures (see Table 1), the stoichiometry and phase composition of the surface of these semiconductors are strongly influenced by temperature and environmental conditions [8,118,119,120]. For example, at normal pressure, the most stable compound, ZnS, does not melt. Under a pressure of 15 MPa (150 atm), ZnS melts at 1850 °C. However, in a vacuum and a neutral atmosphere, this compound can decompose at significantly lower temperatures according to reaction (1) [12].

2A^II^ B^VII^ → 2A^II^_(gas)_ + B_2_^VII^_(gas)_ (1)

For example, according to Singh et al. [121], already at temperatures above 200 °C, the sublimation of sulfur is possible, which at these temperatures has a higher vapor pressure. This means that the surface of ZnS, due to this process, can be enriched with zinc with corresponding changes in its luminescent, adsorption, catalytic, and therefore gas-sensitive properties. In this case, the degree of zinc enrichment will depend on both the temperature and the time of thermal treatment. As is known, the equilibrium vapor pressure of volatile components strongly depends on temperature. It should also be taken into account that the dissociation of II–VI compounds is accompanied by the formation of point defects, which, due to diffusion in the bulk of the material, affect the optical and electrical properties of the compounds.

**Figure 14:**
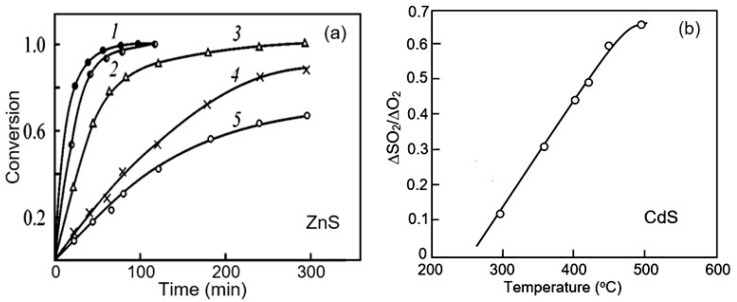
(**a**) Kinetic curves of ZnSe oxidation at the following temperatures: 1—700 °C, 2—600 °C, 3—550 °C, 4—500 °C, and 5—430 °C. Adapted with permission from [126]. Copyright 2001: Springer. (**b**) Relative amount of SO_2_ produced during the reaction of CdS with oxygen versus temperature. A conversion of 1.0 corresponds to 100% transformation of II–VI compounds into the oxide phase. Adapted with permission from [32]. Copyright 1968: Elsevier.

When heating compounds II–VI in air, in addition to their decomposition, the formation of oxides is also possible, since anionic and cationic oxides are more stable than the corresponding selenide, sulfide, or telluride Cd (and Zn) [120]. As can be seen in Figure 14a, already at a temperature of 430 °C, oxidation affects the bulk region of ZnS grains. This suggests that changes on the surface can occur at significantly lower temperatures. This has been confirmed experimentally. For example, according to Bootsma [32], when oxygen and CdS react at temperatures above 250 °C, SO_2_ appears, which is the result of an oxidation reaction (Figure 14). Moreover, this process accelerates with increasing temperature. For example, the processing of ZnS nanoparticles at temperatures up to 400 °C led to the formation of a significant amount of sulfates fixed on the surface of the particles, which was detected by FTIR, Raman scattering, and XPS methods, and at temperatures above 500 °C it led to the complete conversion ZnS to ZnO [122].

**Figure 15:**
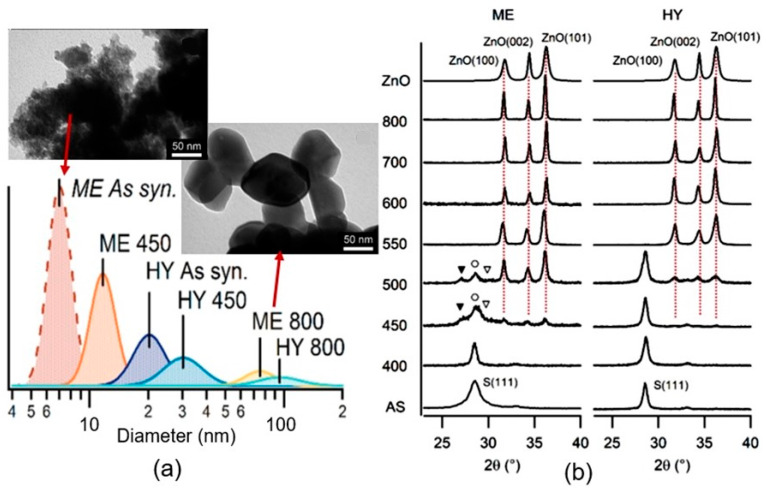
(**a**) Log-normal approximate numerical distribution of ZnS nanoparticle diameter obtained from TEM images. The inserts in the figure are SEM images of ZnS powders after appropriate treatments. (**b**) Evolution of the XRD diffractograms at different calcination temperatures. The S(111) index is referred to as sphalerite-phase ZnS. The dotted lines emphasize the presence of the three main reflections of ZnO. Markers show the presence of the wurtzitic ZnS reflections: (▼) = (100), (○) = (100), and (▽) = (100). AS = as-synthesized. Temperatures are indicated in Celsius degrees. ME—miniemulsion; HY—hydrothermal synthesis. Reprinted with permission from [122]. Copyright 2018: ACS.

The above transformation can be clearly seen in the X-ray diffraction patterns shown in Figure 15b. At the same time, this process was accompanied by the growth of crystallites (Figure 15a), the release of SO_2_, and a change in photocatalytic activity. Although, it should be recognized that changes in the electrical and structural properties of these compounds can begin at lower temperatures [123,124]. It is important to note that due to the preferential interaction of oxygen with S atoms and the subsequent sublimation of SO_2_, the surface of ZnS and CdS is enriched with Cd or Zn metal atoms [25,125]. The same process is characteristic of selenides, since SeO_2_, like SO_2_, has a high vapor pressure (~2 × 10^−6^ Torr).

A similar result was obtained by Shanmugam et al. [127] and Park et al. [73]. They found that annealing at 500 °C in air converts ZnS nanocrystals into ZnO through oxidation. Moreover, it was found that crystallographic modifications of ZnS are oxidized at different temperatures and these processes have different mechanisms. Schulze et al. [128] found that wurtzite oxidation begins at a higher temperature (~560 °C), and ZnO is formed without any detectable by-products or intermediates (Equation (2)). The oxidation of sphalerite begins at a lower temperature (~500 °C), and ZnSO_4_ and Zn_3_O(SO_4_)_2_ are formed intermediately in side reactions with the formation of ZnO (Equations (3)–(6)). It can be seen that the oxidation of sphalerite is a more complex process, including several stages.

*Oxidation of wurtzite:*

ZnS + 3/2O_2_ → ZnO + SO_2_ (2)

*Oxidation of sphalerite*

ZnS + 2O_2_ → ZnSO_4_ (3)

3ZnSO_4_ → Zn_3_O(SO_4_)_2_ + SO_3_ (4)

Zn_3_O(SO_4_)_2_ → 3ZnO + 2SO_3_ (5)

2SO_3_ ↔2SO_2_ + O_2_ (6)

It should be noted that the transformation of ZnS into ZnO at elevated temperatures also occurs in water vapor, even in the absence of oxygen. Sasaoka et al. [129] showed that ZnS can be converted to ZnO, SO_2_, and H_2_ by H_2_O in the absence of O_2_ at high temperature (>600 °C). In the first stage of oxidation, ZnS reacted predominantly with H_2_O. SO_2_ formed from ZnS contains oxygen from H_2_O. Based on the experimental results, it was found that the oxidation of ZnS in the presence of H_2_O can be expressed by the following two equations, Equations (7) and (8):

ZnS + 3H_2_O ⇔ ZnO + SO_2_ + 3H_2_ (7)

3H_2_ + ^3^/_2_O_2_ ⇒ 3H_2_O (8)

**Figure 16:**
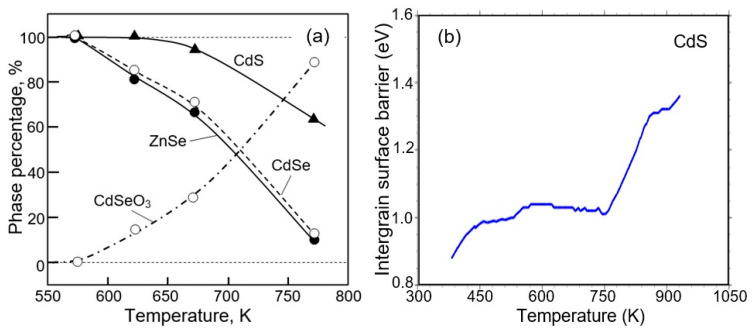
(**a**) The rate of phase conversion of ZnSe, CdSe, and CdS into the oxide phase with temperature. The phase percentage indicates the percentage of II–VI semiconductors and their oxide phase in the heat-treated material. Data extracted from [138]. (**b**) Intergrain barrier measurements for CdS thick film subjected to thermal treatment in a synthetic air atmosphere. All measurements were carried out in a chamber at 25 °C. Reprinted from [40]. Published 2016 by MDPI as open access.

As we indicated earlier, ZnS is the most stable compound. This means that for other less stable II–VI compounds, these decomposition and oxidation processes will begin at even lower temperatures. In particular, as can be seen in Figure 16a, for ZnSe and CdSe at 300 °C, changes in the phase composition of not only the surface but also the bulk of crystallites begin. Moreover, Dengo et al. [122] found that decreasing the size of ZnS crystallites also affects the temperature at which the transition of ZnS to ZnO occurs. For example, Park et al. [74,99] and Yang et al. [130] showed that when the ZnS particle size is reduced to 3–5 nm, complete oxidation of particles occurs at temperatures below 300 °C. The oxidation processes of CdS, CdSe, CdTe, and ZnSe have been studied in sufficient detail in [32,124,125,126,131,132,133,134,135].

Thus, intense oxidation of II–VI compounds at elevated temperatures significantly narrows the range of operating temperatures at which gas sensors will be sufficiently stable over time. Some believe that the most stable compounds for gas sensors, ZnS and CdS, can operate at operating temperatures up to 250–300 °C [40,62,136,137]. But these conclusions are not supported by the results of long-term testing of sensors in such conditions. There are simply no such studies. In most studies, the testing time for sensors operating at temperatures of 250–300 °C was limited to no more than 20–30 min [38,84]. Gaiardo et al. [40] analyzed the change in electrical conductivity of CdS sensors during heat treatment and estimated the change in the height of the potential barrier at the grain boundary. They found that a change in the height of the potential barrier between CdS grains due to oxidation occurs already at the lowest temperatures (see Figure 16b), increasing significantly at T > 500 °C, when oxidation affects not only the surface but also the bulk region of the CdS grain. As is known, it is the potential barrier at the grain boundary that controls conductivity in polycrystalline materials.

The accelerated decomposition and oxidation of II–VI compounds with increasing temperature also excludes the possibility of using high-temperature annealing, which is often used in metal oxide sensors to restore the original surface properties after long-term operation [139,140]. Of course, through long-term aging, it is possible to stabilize the parameters of gas sensors based on II–VI compounds operating at high temperatures. But in this case, the gas-sensitive properties will be determined not by the properties of II–VI semiconductors but by the properties of the metal oxides formed on their basis. At that, it should be understood that the gas-sensitive parameters of such sensors will differ significantly from the parameters of the initial sensors based on II–VI compounds. For example, Gaiardo et al. [40] compared the performance of CdS and CdO sensors and found that CdO sensors had a negligible conductometric response to ethanol compared to CdS sensors.

In order to eliminate or significantly slow down the oxidation process of II–VI compounds, many studies have proposed the use of these compounds to develop sensors operating at room temperature [24,105,141,142,143] and to increase their sensitivity in the low-temperature region, using illumination [40,144]. Of course, lowering the operating temperature slows down the degradation processes associated with oxidation [32], and sensors based on II–VI compounds can indeed function without noticeable changes in parameters for a longer time. For example, the response of CdS nanoflakes to 200 ppm isopropanol was found to be stable with little change in response over 20 days [58]. Further testing of CdS-based sensors demonstrated even longer periods of stability, ranging from 42 to 60 days, when exposed to gases such as ethanol [40], some volatile organic compounds [145], and LPG [40,146]. Tests on ZnS gas sensors exposed to LPG have also shown good stability when operated at room temperature for 30 days [147]. However, Navale et al. [54] reported that CdS sensors designed to detect NO_2_ at 38 °C require an aging period (~20 days) before the sensor response enters a stable period.

**Figure 17:**
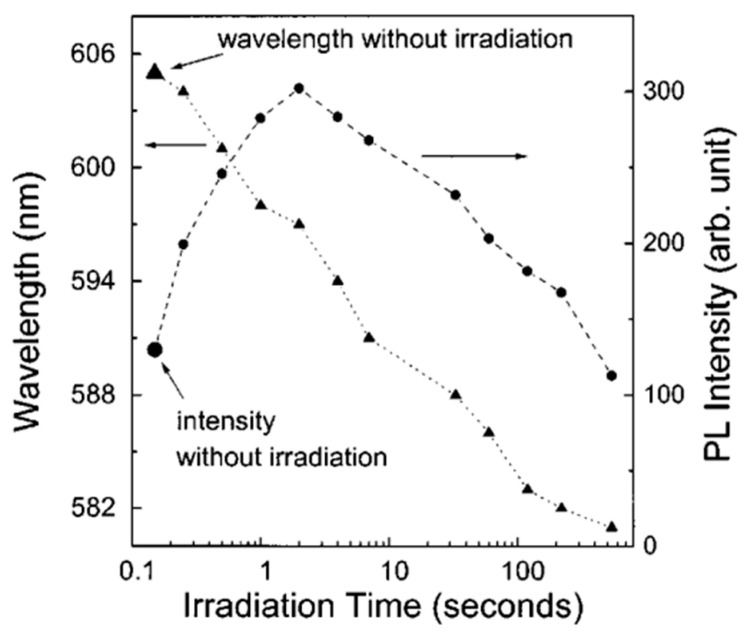
Intensity and peak wavelength position of the PL spectra vs. the photoirradiation time for the CdSe QDs. Reprinted with permission from [149]. Copyright 2003: AIP Publishing.

But experiments have shown that oxidation, although at a lower rate, also occurs at low temperatures [125,132,133], and illumination, especially UV irradiation, stimulates this process [148,149]. It is the oxidation of the semiconductor material, accompanied by the formation of an oxide layer and traps at grain boundaries, that causes a decrease in the mobility of charge carriers and a decrease in the conductivity of polycrystalline II–VI semiconductor films [150]. It was found that the thickness of the oxide phase on the surface of II–VI compounds depends on the state of the surrounding atmosphere and the treatment to which these compounds were subjected. For example, according to Yi and Liu [151], the TeO_2_ layer on the surface of a polycrystalline CdTe film after exposure to air at room temperature for 6 months has a thickness of ~2 nm. Zazvorka et al. [152] investigated the growth of the surface oxide layer on the surface of CdTe and CdZnTe crystals after chemical etching in bromine solutions and found that rapid growth was visible within five days after chemical treatment, followed by half-saturation and a decrease in the growth rate of the oxide layer after the first weeks. After a month, an oxide layer about 3 nm thick appeared on all samples. It was found that this effect is most pronounced for small QDs, in which the area subject to oxidation becomes comparable to the diameter of these QDs. For example, according to Hu and Wu [148], the photocorrosion effect decreased the size of CdSe quantum dots and caused a blue shift of the band edge (BE) peak. At the same time, they noticed that under the same UV irradiation, the PL spectrum of small QDs changes faster than the PL spectrum of large QDs. The same effect was observed by Wang et al. [149]. These results are shown in Figure 17. CdSe QD samples with a diameter of ~4.5 nm were illuminated with laser radiation at a wavelength of 420 nm. It is important to note that irradiation with 420 nm laser pulses had no obvious effect on the CdSe QDs when the sample was placed in a vacuum. However, if the sample was exposed to ambient air, photoirradiation caused a blue shift in the PL peak position and a significant change in the PL intensity. If we take into account that reducing the size of crystallites is one of the most effective methods for increasing the sensory response of conductometric gas sensors [15], then the tendency of small crystallites of II–VI compounds to rapidly oxidize in an oxygen atmosphere limits the use of this approach to improve the parameters of sensors with the basis of these semiconductors. In photodetectors based on II–VI compounds, the aging process is combated through passivation and surface encapsulation using various coatings (SiO_2_, SiN_x_, Al_2_O_3_, etc.) with excellent insulating properties [153,154,155,156]. But in gas sensors, this approach is unacceptable.

✓ It is important to note that studies have shown that II–VI compounds have more complex surface chemistry [157]. As is known, metal oxides do not change their phase state in an oxygen atmosphere. While only hydroxides can form on the surface of metal oxides in a humid atmosphere [158], metal hydroxides, oxides, sulfur dioxide, and sulfates can be observed on the surface of ZnS and CdS when heated in the atmosphere [124,128], the ratio of which will depend on temperature and air humidity. These CdS oxidation products and the corresponding reactions in a dry atmosphere that can lead to the formation of indicated compounds are listed in Table 3. The possibility of the formation of hydroxyls on the surface of II–VI compounds was shown by Hellgren et al. [159] using ZnSe as an example. They found that the oxide composition on the ZnSe surface is indeed very sensitive to the surface treatment method, and in the presence of water or water vapor, hydroxyls such as SeOH and ZnOH can be formed on the surface.

**Table 3:** Chemical reactions that occur during the thermal oxidation of CdS.

No.	CdS Oxidation Product	Chemical Reaction Equation
1	CdSO_4_	CdS + 2O_2_ → CdSO_4_
2		Cd_3_SO_6_ + 2SO_2_ + O_2_ → 3CdSO_4_
3		Cd_5_S_3_O_6_ + 2SO_2_ + 5O_2_ → 5CdSO_4_
	CdO	3CdS + 5O_2_ → 2CdO⋅CdSO_4_ + 2SO_2_
4		2CdS + 3O_2_ → 2CdO + 2SO_2_
5		CdS + CdSO_4_ → 2CdO + 2SO_2_
6		CdSO_4_ → CdO + SO_2_ + 1/2O_2_
7	Cd_3_SO_6_	6CdO + 2SO_2_ + O_2_ → 2Cd_3_SO_6_
8	Cd_5_S_3_O_6_	5CdSO_4_ → Cd_5_S3O_6_ + 2SO_2_ + 5O_2_
9		5CdS + 5O_2_ → Cd_5_S_3_O_6_ + 2SO_2_
10		5Cd_3_SO_6_ + 4SO_2_ → 3Cd_5_S_3_O_6_ + 10O_2_

*Source*: Data extracted from [119,124,160,161,162,163].

**Figure 18:**
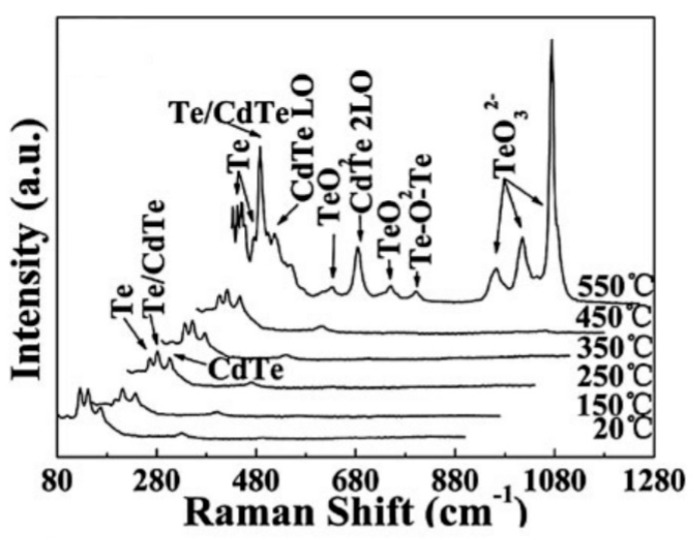
Raman spectra of CdTe films after annealing at different temperatures (recorded at room temperature). Reprinted with permission from [131]. Copyright 2012: Wiley.

In the case of tellurides, the situation can be even more complicated. Thus, according to Bai and Wang [131], when thin CdTe films are annealed at temperatures up to 550 °C, the main oxides formed on the surface are CdTeO_3_, TeO_2_, and Te_2_O_5_ [131]. In addition to oxides, Te was also observed after annealing, resulting from the decomposition of CdTe at high annealing temperatures (Figure 18). This result is quite reasonable since TeO_2_ is more stable than CdO. In addition, according to thermodynamic data [14], the vapor pressure of TeO_2_ is very low (less than 10^−28^ Pa) at room temperature.

In the case of ZnTe, TeO_2_ dominates the surface only at a low surface oxygen coverage (θ ≤ 0.5 ML), whereas at a high oxygen coverage (θ > 1 ML), ZnO appears at the surface [25,132]. This means that in reality, the top layer of ZnTe usually contains both TeO_2_ and ZnO. The lack of dominance of TeO_2_ in the top layer was explained by the fact that ZnO is more stable than TeO_2_ and the reaction (Equation (9)). According to this reaction, TeO_2_ is reduced by Zn to a lower oxide, which sublimates from the surface, leaving ZnO on the surface [25].

TeO_2_ + Zn→ TeO↑ + ZnO (9)

**Figure 19:**
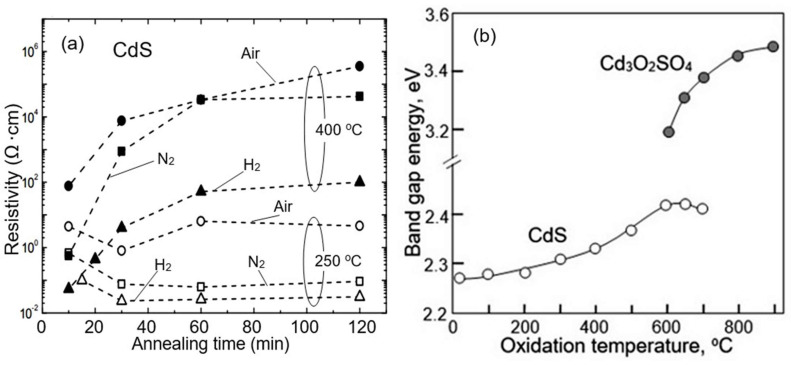
(**a**) Electrical resistivity of CdS films at RT as a function of annealing time, temperature, and atmosphere (H_2_, N_2_, and air). Adapted with permission from [123]. Copyright 2014: Elsevier. (**b**) Change in the band gap of CdS samples during their oxidation. Reprinted with permission from [124]. Copyright 2019: RSC Publishing.

**Figure 20:**
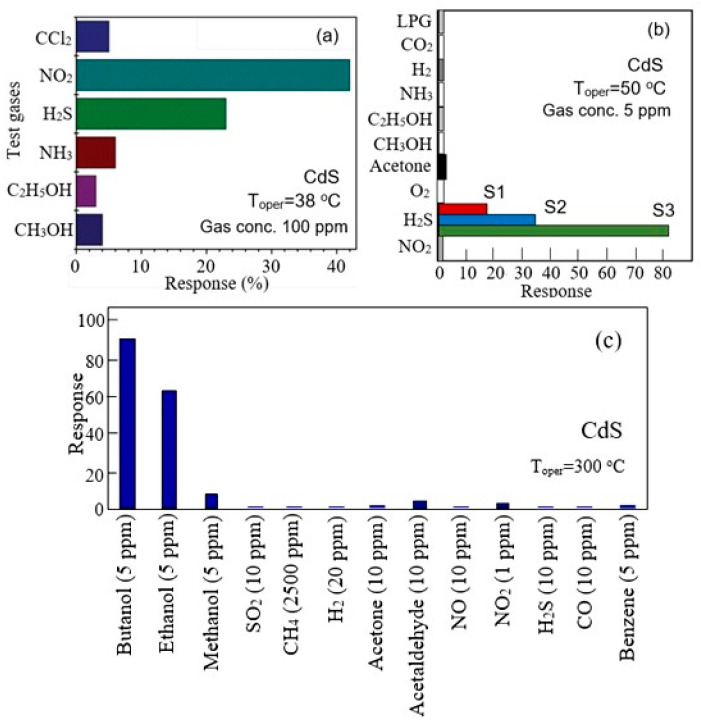
Responses of CdS sensors developed in various laboratories to the tested gases: (**a**) CdS was prepared using the chemical bath deposition method (pH = 10–11). Reprinted with permission from [54]. Copyright 2014: RSC. (**b**) CdS films were deposited by chemical bath deposition from solutions with different pH: S1-pH = 9; S2-pH = 9.5, and S3-pH = 10. Reprinted from [51]. Published 2016 by SCISET LLC as open access. (**c**) CdS was synthesized as nanoparticles via precipitation reactions in aqueous solution. Reprinted from [40]. Published 2016 by MDPI as open access.

Ambiguity and uncertainty in the state of the surfaces of II–VI semiconductors create difficulties both in interpreting processes occurring on their surface and in predicting the results of the influence of technological operations on the parameters of developed gas sensors. As shown in [163], technological operations using heat treatments (300–500 °C) in various environments, in addition to changing the phase composition of films, affect the crystallite size, dislocation density, the likelihood of defect formation packaging, electrical conductivity (Figure 19a), and the band gap of the semiconductor (see Figure 19b). All these changes naturally affect the gas sensors’ performances, since it is these material parameters that largely control gas-sensing effects [15]. For example, an increase in the concentration of Cd on the surface of CdS is accompanied by a change in the concentration of chemisorbed oxygen, which is involved in gas-sensing effects [64]. A clear example of the influence of the prehistory of compounds II–VI used in the manufacture of gas sensors on the parameters of these sensors is the huge variation in the characteristics of sensors manufactured in different laboratories (see Figure 20). When using the same semiconductor, sensors can exhibit increased sensitivity to different gases, and the sensory response to the same gas can differ by several orders of magnitude [51,54].

✓ It is important to note that chemical processing also affects the surface stoichiometry of II–VI compounds, which further complicates the process of comparing sensors prepared in different laboratories [157]. This also makes it difficult to achieve the required reproducibility of sensor parameters. For example, fresh Br_2_/ethanol solutions have been reported to form a nearly stoichiometric CdSe surface slightly depleted in cadmium, as evidenced by a Cd:Se ratio of 0.91 as determined by X-ray photoelectron spectroscopy (XPS). In contrast, aged solutions (~5 h) resulted in the formation of a CdS surface rich in Cd (Cd:Se ≈ 1.6 by XPS) [45,164,165]. Jaseniak and Mulvaney [165] demonstrated that CdSe nanocrystals with Cd- or Se-rich surfaces have significant differences in chemical nature, adsorption properties, and stability. The results of the effect of chemical treatment on the surface stoichiometry of ZnSe are given in Table 4 [166].

**Table 4:** Effect of etching on the surface stoichiometry of ZnSe.

Etchant	Etchant Composition	ZnSe
		Se/Zn
1	1% Bromine in methanol	1.84
2	1 g of K_2_Cr_2_O_7_ in 10 mL H_2_SO_4_ + 20 mL H_2_O	1.21
3	Ar^+^ ion bombarded for ZnSe (ref. surfaces)	0.72
4	0.5 g of NaOH + 0.5 g of Na_2_S_2_O_3_ in 100 mL H_2_O	0.69
5	Etchant 2 followed by etchant 4	0.68
6	Etchant 1 followed by a wash in hot NaOH solution (80–90 °C)	0.58
7	1 g of NaOH in 20 mL H_2_O + 1 mL H_2_O_2_	0.57
8	Etchant I followed by hydrazene-hydrate	-

*Source*: Data extracted from [166].

How critical the choice of chemical processing is in the sensor manufacturing process can also be determined based on the results reported by Reese et al. [167]. They found that CdTe samples with a Te-rich surface exhibited significant temporal instability, whereas samples with a stoichiometric or Cd-rich surface were more stable. This means that when preparing a CdTe surface, the use of treatments (see Table 5) that promote the formation of a Te-rich surface should be avoided. Dharmadasa et al. [166,168,169] also found a strong influence of CdTe surface stoichiometry on the height of the potential barrier at Schottky contacts. They found that Te-rich surfaces tend to have lower Schottky barriers (~0.74 eV), whereas Cd-rich surfaces tend to have higher Schottky barriers (~0.93 eV). The Te-enriched surface was obtained by etching in a bromine/methanol solution, and the Cd-enriched surface was obtained by etching in an oxidizing agent followed by etching in an alkaline solution. This change in the Schottky barriers indicates a strong change in the electronic properties of the surface, which directly affects gas-sensitive effects.

As shown in [43], chemical treatment also stimulates the formation of an oxide layer on the surface of II–VI compounds. For example, it was found that after etching CdSe under the influence of air, a rapid formation of surface oxide occurs. From ellipsometric measurements of CdSe, it was found that the oxide layer on the Se-rich side is approximately three times thicker than on the Cd-rich side, suggesting that the Se-rich surface is more susceptible to oxygen attack. Interestingly, the thickness of the oxide layer on the Se-rich surface was correlated with the etching duration, with thicker oxide obtained when using long (>5 min) etching.

## 4. Summary

The analysis showed that II–VI semiconductors have properties suitable for the development of highly sensitive gas sensors, which has been repeatedly demonstrated using the example of conductometric sensors capable of detecting various toxic and flammable gases and vapors. However, despite this, it must be recognized that these sensors should be considered only as an object for studying the nature of gas-sensitive effects and not as sensors intended for the market. Developing gas sensors for the market requires an approach that is fundamentally different from that often used in published papers. For the sensor market, the main parameter of a sensor is not the magnitude of the sensory response but the reproducibility of sensor parameters during their manufacturing process and the stability of their characteristics during their entire service life, which can be several years. It is these factors that hinder the advancement of II–VI semiconductor-based gas sensors to the market. Unfortunately, this drawback is fundamental and it is not possible to eliminate it using available methods. The lack of comprehensive studies in the field of II–VI semiconductor-based conductometric gas sensors is also regrettable.

## Figures and Tables

**Table 1:** Parameters of chalcogenides used in gas sensors.

Semiconductor	E_g_ (eV)	T_melting_ (°C)	T_sublimation_ (°C)	T_oxidation_ (°C)
ZnS	3.54–3.91	1827–1850	1178	<500
ZnSe	2.7–2.721	1525	800–900	<300
ZnTe	2.25–2.27	1238–1290	~600	<300
CdS	2.42–2.46	1475–1750	980	<450–500
CdSe	1.74–1.751	1268	600–700	<300
CdTe	1.49–1.51	1092	~500	<300

**Table 2:** Sensing properties of the Zn_1−x_Cd_x_S (X = 0−1.0) nanowire-based sensors to 20 ppm ethanol at 206 °C.

Parameter	Material (Zn_1−x_Cd_x_S)					
	X = 0	X = 0.2	X = 0.4	X = 0.6	X = 0.8	X = 1.0
Response (R_a_/R_g_)	2.1	7.5	12.8	11.3	9.8	6.6
Rise time (s)	6	1	2	2	<1	<1
Recovery time (s)	8	1	1	1	1	<1

*Source*: Reprinted with permission from [55]. Copyright 2015: ACS.

**Table 5:** Summary of surface compositions of CdTe extracted by XPS of various samples examined by Reese et al. [154].

Sample Treatment	Cd/Te
As-received	0.59
Br_2_:MeOH	0.73
3 kV Ar^+^	1.08
Atomic H	1.10
Ar^+^, 250 °C UHV	1.09
Cd ampoule anneal	1.29
Te ampoule anneal	1.05
UV/O_3_	1.81
NaOH:Na_2_S_2_O_4_	1.35
KOH	1.12
Hydrazine	0.86
CH_3_COOH:HNO_3_:H_2_SO_4_	0.004
FeCl_3_	0.03
HNO_3_:K_2_Cr_2_O_7_:Cu(NO_3_)_2_	0.08
HNO_3_:K_2_Cr_2_O_7_	0.06

*Source:* Data extracted from [167].

## Data Availability

Not applicable.
